# Neuroprotetive potential of *Taraxacum officinale* leaf extract against cisplatin neuropathy via antioxidative modulation

**DOI:** 10.1055/s-0045-1813237

**Published:** 2025-12-08

**Authors:** Mehmet Erdem, Hasan Ulusal, Mehmet Özaslan

**Affiliations:** 1 Gaziantep University, Department of Health Services Vocational School, Gaziantep, Türkiye.; 2 Gaziantep University, Department of Biology, Gaziantep, Türkiye.; 3 Gaziantep Islam Science and Technology University, Department of Medical Biochemistry, Gaziantep, Türkiye.

**Keywords:** Cisplatin, Peripheral Nervous System Diseases, Taraxacum, Oxidative Stress, Antioxidants, Enzymes, Neuroprotection

## Abstract

**Background:**

Cisplatin, a potent platinum-based chemotherapeutic, effectively treats various cancers. However, it is limited by cisplatin-induced peripheral neuropathy (CIPN), driven by oxidative stress and neuroinflammation.

**Objective:**

The present study investigates the neuroprotective potential of *Taraxacum officinale* L. leaf extract (TOE), rich in polyphenols such as luteolin and quercetin, known for their antioxidant and antiinflammatory properties.

**Methods:**

Molecular docking of 10 polyphenols against NF-κB1 revealed that luteolin and quercetin outperform synthetic inhibitors, forming strong interactions with key residues. These compounds exhibited favorable pharmacokinetics, including high gastrointestinal absorption and nontoxicity. The CIPN was induced in male Wistar albino mice (3 mg/kg cisplatin, i.p., weekly for 5 weeks), with TOE (500 mg/kg, intragastric, daily) or saline administered concurrently. A TOE-only group served as a control. Behavioral assessments (rotarod, hot plate, cold plate, tail flick) evaluated sensory and motor function, while biochemical assays measured antioxidant enzymes (CAT, GPx1, SOD2), oxidative stress markers (MDA, TOS, IMA), and proinflammatory cytokines (NF-κB, TNF-α, IL-6) in serum and sciatic nerve tissues.

**Results:**

Cisplatin induced significant behavioral deficits, reduced antioxidant capacity, and elevated oxidative and inflammatory markers. The TOE significantly ameliorated these effects, restoring behavior, enhancing antioxidant status, and reducing inflammation, consistent with the in silico predictions of NF-κB1 inhibition.

**Conclusion:**

These findings highlight *T. officinale* as a promising, safe, complementary therapy for CIPN, warranting further clinical exploration.

## INTRODUCTION

Cisplatin (CIS), a cornerstone platinum-based chemotherapeutic, is widely used to treat malignancies such as ovarian, testicular, and lung cancers. 1 However, its clinical utility is constrained by dose-dependent neurotoxic side effects, particularly CIS-induced peripheral neuropathy (CIPN), which affects 60 to 70% of patients. 2 This condition manifests as sensory and motor impairments, including paresthesia, dysesthesia, and neuropathic pain, driven by oxidative stress from excessive reactive oxygen species (ROS) and neuroinflammatory pathways involving nuclear factor-kappa B (NF-κB), tumor necrosis factor-alpha (TNF-α), and interleukin-6 (IL-6). 3 4 Current treatments, such as gabapentin and duloxetine, offer limited efficacy and often introduce adverse effects, underscoring the need for novel, safe interventions. 5 6

Phytochemicals with antioxidant and anti-inflammatory properties have emerged as promising candidates for CIPN management. *Taraxacum officinale* L., commonly known as dandelion, is a medicinal plant rich in polyphenols, notably luteolin and quercetin, which exhibit potent antioxidant and antiinflammatory effects through NF-κB inhibition and enhancement of antioxidant enzymes, like catalase (CAT), glutathione peroxidase (GPx1), and superoxide dismutase (SOD2). 7 8 9 While *T. officinale* has shown therapeutic potential in conditions such as hepatic injury and neuroinflammation, 10 11 its role in ameliorating CIPN remains underexplored, despite the urgent need for targeted therapies to improve patient quality of life and treatment adherence. 12 13

This study evaluates the neuroprotective effects of *T. officinale* leaf extract (TOE) in a mouse model of CIPN, focusing on its modulation of oxidative stress and NF-κB-mediated inflammatory pathways. By integrating in silico molecular docking of polyphenols against NF-κB1, in vivo behavioral assessments, and biochemical analyses of oxidative and inflammatory markers, this investigation elucidates the mechanistic basis of the extract's therapeutic potential and assesses its suitability as a complementary treatment for CIPN.

## METHODS

### Plant collection, extraction, and polyphenol profiling

Fresh leaves of *T. officinale* L. (Asteraceae) were collected during the vegetative stage (April–May 2023) from Gaziantep, Turkey. Plant material was authenticated by a botanist at Gaziantep University, with a voucher specimen (TU-2023-01) deposited in the university's herbarium.

The leaves were washed with deionized water and shade-dried at room temperature, then pulverized. There were 50 g of powdered leaf material, macerated in 1 L of 96% ethanol for 14 days at room temperature with periodic agitation to optimize polyphenol extraction. 14 The mixture was filtered using Whatman (Global Life Sciences Solutions USA LLC.) No. 1 filter paper, and the solvent was evaporated under reduced pressure via rotary evaporation (Heidolph Instruments GmbH & Co. KG). The extract yield was approximately 12% w/w and was standardized for key polyphenols (e.g., luteolin, quercetin) using high-performance liquid chromatography (HPLC) to ensure batch consistency and guide molecular docking. 14

Extracts were stored at 4°C and reconstituted in sterile saline for in vivo use. All steps were performed protected from light and the reconstituted extract was prepared fresh daily to ensure stability. Therefore, the ethanolic extract was not fractionated to preserve synergistic polyphenol effects, being standardized by HPLC instead, to confirm the presence of key neuroprotective polyphenols (e.g., luteolin, quercetin) for batch consistency, without quantitative profiling as the study prioritized whole-extract efficacy in CIPN.

### Protein modeling and ligand preparation

The NF-κB1 protein sequence (p105 subunit) from *Mus musculus* (UniProt ID: P25799) was retrieved from the NCBI database, selected for homology with mouse NF-κB1 relevant to the CIPN model. 15 Homology modeling was conducted using a SWISS-MODEL (Computational Structural Biology Group) with the 1NFK_A template for its high sequence identity and structural resolution. 16 The physicochemical properties ( **Supplementary Material 1** , available at - available at https://www.arquivosdeneuropsiquiatria.org/wp-content/uploads/2025/09/ANP-2025.0265-Supplementary-Material.docx , Table 1 ), including molecular weight and isoelectric point, were calculated using the ProtParam tool on ExPASy (Swiss Institute of Bioinformatics). 17

**Table 1:** The quantitative analysis of oxidative stress indicators and proinflammatory cytokines in serum samples

Parameters	Baseline (mean ± SEM)	CIS-induced (mean ± SEM)	TOE (500 mg/kg) (mean ± SEM)	CIS + TOE (mean ± SEM)	% Change (TOE + CIS vs. CIS)
**NF-κB (ng/mL)**	24.19 ± 1.45	26.83 ± 1.70*	22.14 ± 1.35 ^#^	23.50 ± 1.23 ^#^	−12.4%
**TNF-α (ng/L)**	600.56 ± 6.32	608.78 ± 1.91*	597.78 ± 3.94 ^#^	598.88 ± 5.07 ^#^	−1.6%
**IL-6 (ng/L)**	15.55 ± 0.54	18.76 ± 1.16**	13.30 ± 1.14 ^###^	14.79 ± 0.67 ^###^	−21.2%
**IMA (ng/mL)**	183.92 ± 4.55	203.87 ± 4.75*	176.58 ± 3.47 ^#^	197.19 ± 4.02 ^#^	−3.3%
**MDA (nmol/mL)**	9.64 ± 0.65	11.57 ± 1.80*	8.92 ± 1.15 ^#^	9.15 ± 0.72 ^#^	−20.9%
**TOS (µmol H₂O₂ Eq./g protein)**	12.63 ± 1.51	15.27 ± 1.13*	10.39 ± 0.99 ^###^	12.69 ± 1.22 ^#^	−16.9%
**TAS (mmol Trolox Eq./g protein)**	1.65 ± 0.12	1.24 ± 0.16*	1.72 ± 0.09 ^#^	1.55 ± 0.21 ^#^	+25.0%
**OSI**	7.65 ± 0.26	12.31 ± 0.14**	6.04 ± 0.23 ^###^	8.19 ± 0.28 ^###^	−33.5%

Abbreviations: CIS, cisplatin; IL-6, interleukin-6; IMA, ischemia-modified albumin; OSI, oxidative stress index; SEM, standard error measurement; MDA, malondialdehyde; NF-κB, nuclear factor-kappa B; TAS, total antioxidant status; TNF-α, tumor necrosis factor-alpha; TOE, *T. officinale* leaf extract; TOS, total antioxidant status.

Note: OSI calculated as TOS/TAS ratio. Statistical significance: *control vs. CIS *p* < 0.05; **control vs. CIS *p* < 0.001; ^#^ CIS vs. TOE + CIS: *p* < 0.05; ^###^ CIS vs. TOE + CIS: *p* < 0.001.

A library of 10 polyphenols from *T. officinale* (luteolin, quercetin, caffeic acid, p-coumaric acid, apigenin, kaempferol, luteolin-7-O-glucoside, chlorogenic acid, 3,5-dicaffeoylquinic acid, quercetin-3-O-glucoside) was selected based on antioxidant and anti-inflammatory properties. 18 Parthenolide and Bay 11-7082 were used as reference NF-κB1 inhibitors. Ligand three dimensional (3D) structures were retrieved from PubChem (National Library of Medicine) and PhytoHub (phytohub.eu), then optimized using the 2021 BIOVIA Discovery Studio (Dassault Systèmes) via energy minimization and 3D protonation for stability. 19 Prior to docking, ligand geometries were checked for valence and tautomeric consistency, and duplicates were removed.

### Receptor preparation, docking, and ADME-Tox profiling

The NF-κB1 receptor modeling included removal of water molecules and ligands using Discovery Studio, followed by energy minimization and 3D protonation. 20 Docking simulations were performed using PyRx (Open Source) with AutoDock Vina (The Scripps Research Institute). 21 The active site was defined based on conserved residues in the p50 subunit. 15 Docking was executed with an exhaustiveness parameter of 8 and a grid box that encompassed the GLN-306/ARG-305/LYS-272 region. The lowest-energy pose per ligand was retained for interaction analysis.

Top-scoring polyphenols were evaluated using SwissADME (Swiss Institute of Bioinformatics) 22 to assess gastrointestinal absorption and compliance with Lipinski's rule of five. Acute oral toxicity was predicted using ProTox-II (Charite University of Medicine), classifying compounds into six toxicity classes. 23 Class VI (LD _50_ > 5,000 mg/kg) indicated nontoxic profiles, confirming extract safety at 500 mg/kg for in vivo testing. Computational assessments were used solely to support dose selection and did not replace empirical safety monitoring. While in vitro dose-response analyses could further validate toxicity, they were not performed as the study focused on in vivo neuroprotective efficacy, supported by computational predictions and prior safety data.

### In Vivo studies and treatment design

A total of 28 male Wistar Albino mice (8–10 weeks, 250–300 ± 5g) were obtained from Gaziantep University's Animal Research Center. Mice were housed in ventilated polycarbonate cages under controlled conditions (21 ± 2°C, 50–60% humidity, 12h light/dark cycle) with ad libitum access to pellet feed (DSA Agrifood Product Inc.) and water. Acclimatization lasted 10 days. Ethics approval was granted by Gaziantep University's Animal Ethics Committee, under the protocol No. 2022/257, complying with EU Directive 2010/63/EU and Animal Research: Reporting of In Vivo Experiments (ARRIVE) guidelines.

A CIPN model was adapted from Wang et al. 24 Mice were randomized into four groups (n = 7):

- Control (1 mL saline, intraperitoneal [i.p.], weekly);
- CIS (3 mg/kg/week, i.p., with saline for nephrotoxicity mitigation);
- CIS + *T. officinale* (3 mg/kg/week CIS, i.p.; 500 mg/kg extract in 0.5 mL/100 g body weight saline, intragastric, daily);
- *T. officinale* only (500 mg/kg extract in 0.5 mL/100 g body weight saline, intragastric, daily).

The 500 mg/kg dose was selected based on prior studies demonstrating anti-inflammatory and neuroprotective efficacy in mice model of neurotoxicity at this level without adverse effects, 10 15 25 supported by in silico absorption, distribution, metabolism, excretion, and toxicity (ADMET) profiling indicating high safety margins for the extract's key polyphenols ( Table 2 ). Therefore, serum levels of extract compounds were not measured for pharmacokinetics, as the focus was on neuroprotective endpoints, with bioavailability inferred from in silico ADMET data ( Table 2 ). Treatment spanned 5 weeks, with injections between 12:00 and 13:00. Serum creatinine was monitored posttreatment using blood collected under ketamine/xylazine anesthesia (80/10 mg/kg, i.p.), clotted for 30 minutes, and centrifuged at 3,000 × g for 10 minutes. 24 Serum creatinine levels were within normal ranges across all groups, with a mean ± standard error of measurement (SEM) of approximately 0.5 to 0.7 mg/dL ( *p* > 0.05; data not shown), confirming no confounding nephrotoxicity in the CIPN model. Animal allocation was performed by an investigator not involved in outcome assessments. Body weight and general condition were monitored on weekly basis.

**Table 2:** ADMET-Tox analysis demonstrated drug-like properties of top 10 polyphenol compounds by using Lipinski rules

PubChem ID	Name	MW	H-Bond (acceptor)	H-Bond (donor)	LogP (o/w)	Lipinski roles	Tox. Class
5280445	Luteolin	286.24	5	4	2.28	Yes	VI
689043	Caffeic acid	180.16	4	3	1.2	Yes	VI
5280343	Quercetin	302.24	6	5	1.99	Yes	VI
637542	p-Coumaric acid	164.16	3	2	1.49	Yes	VI
5280443	Apigenin	270.24	4	5	2.58	Yes	VI
5280863	Kaempferol	286.24	5	4	2.28	Yes	VI
5280637	Luteolin-7-O-glucoside	448.38	10	7	−0.24	No	VI
1794427	Chlorogenic acid	354.31	9	6	−0.65	No	VI
5280633	3,5-Dicaffeoylquinic acid	354.31	9	6	−0.65	No	VI
5280804	Quercetin-3-O-glucoside	464.38	11	8	−0.54	No	VI

Abbreviation: ADMET, absorption, distribution, metabolism, excretion, and toxicity.

Note: Analysis performed using SwissADME and ProTox-II. All compounds meet Lipinski's rule (MW < 500 Da, H-bond donors ≤ 5, H-bond acceptors ≤ 10, LogP < 5) unless noted. Toxicity Class VI indicates LD50 > 5,000 mg/kg (non-toxic).

### Behavioral and biochemical assessments

On day 36, blinded investigators conducted behavioral tests: RotaRod (Ugo Basile, motor coordination), hot plate (thermal sensitivity), cold plate (cold hyperalgesia), and tail flick (nociceptive threshold), with 24-hour intervals to minimize stress. RotaRod was set at 5 to 40 rpm, 300 s cut-off; the mean of three trials was used with 5 min rests. 25 Cold and hot plate assays were conducted at 5°C (50 s cut-off) and 55°C (30 s cut-off), respectively. 4 Tail flick response was measured at a 10 s cut-off. 26 Animals were habituated to the testing room for ≥ 30 min before each assay to reduce stress-related variability, and surfaces were cleaned between trials.

At study endpoint, around 5 mL of blood was collected via cardiac puncture under ketamine/xylazine anesthesia (80/10 mg/kg, i.p.). Serum was obtained by 30 minute clotting at room temperature and centrifugation at 2,000 × g for 10 minutes at 4°C. Plasma was collected into ethylenediaminetetraacetic acid (EDTA) tubes. Bilateral mid-thigh sciatic nerve segments were harvested; epineural sheaths were removed under a stereomicroscope. Nerves were homogenized in PBS (pH 7.4) with protease inhibitors using a Polytron homogenizer (Polytron), then centrifuged at 12,000 × g for 15 min at 4°C. Protein concentrations were quantified with the Taken3 apparatus on an H1 Synergy device (Biotek). All biochemical measurements were performed by personnel blinded to group allocation.

Antioxidant enzymes (CAT, GPx1, SOD2) and oxidative stress markers (malondialdehyde [MDA], total oxidant status [TOS], ischemia-modified albumin [IMA]) were quantified using ELISA kits (Bioassay Technology Laboratory; CAT: E0869Ra, GPx1: E01172Ra, SOD2: E2268Ra, MDA: E0156Ra, IMA: E0638Ra). Proinflammatory cytokines (TNF-α, IL-6, NF-κB) were measured using enzyme-linked immunosorbent assay (ELISA) kits (TNF-α: E0764Ra, IL-6: E0135Ra, NF-κB: E0287Ra), verifying in silico predictions. The total antioxidant status (TAS) was determined spectrophotometrically. Results were normalized to total protein concentration. 7 15

All ELISAs were run in duplicate with manufacturer-provided standards and internal controls. Plates with CV > 10% were repeated. So, serum was used for all biochemical assays (e.g., ELISA for cytokines and antioxidants, spectrophotometry for TAS), while plasma was collected but not utilized in the presented analyses. Additionally, ELISA was selected over methods like Western blotting for its quantitative precision in measuring enzyme levels, given the study's emphasis on functional antioxidant modulation in neuroprotection.

### Statistical analysis

Data were presented as mean ± SEM. The two-way analysis of variance (ANOVA) was used to evaluate treatment and time effects, followed by Tukey's post-hoc test, through the IBM SPSS Statistics for Windows (IBM Corp.), version 25. Normality and homogeneity of variances were assessed using Shapiro-Wilk and Levene's tests. Statistical significance was set at *p* < 0.05. Pairwise comparisons were denoted as control versus CIS (*) and CIS versus treatment groups (#).

## RESULTS

### In silico analysis of NF-κB1 inhibition by *T. officinale* polyphenols

In silico analysis identified TOE polyphenols as potential NF-κB1 inhibitors. The NF-κB1 p105 subunit (971 amino acids; UniProt: P25799) was validated as the docking target via SWISS-MODEL. Ramachandran plots ( Figure 2A and 2B ) and structural metrics, with the global model quality estimate (GMQE: 0.73, 100% query coverage, 87.75% similarity; **Supplementary Material 1 Table S1A and Table S1B** ), confirmed the reliability of the model.

**Figure 1:**
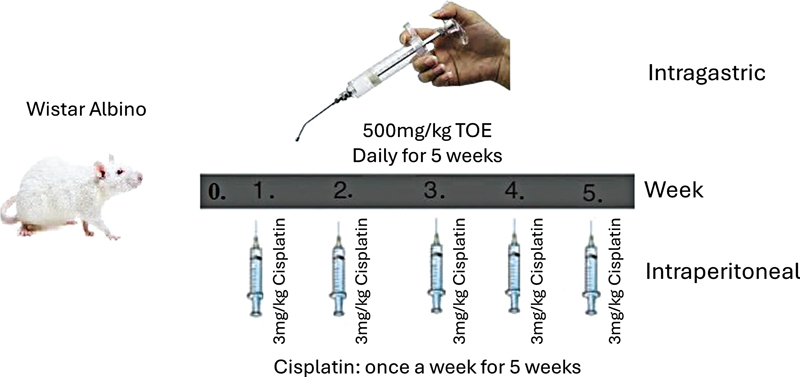
Schematic representation of experimental design using Wistar albino mice. The experimental design involved four groups of male Wistar albino mice (n = 7 per group): Control (normal saline, 1 mL, i.p., weekly), CIS (3 mg/kg, i.p., weekly), *T. officinale* extract (TOE, 500 mg/kg, intragastric, daily), and CIS + TOE (3 mg/kg CIS i.p. weekly + 500 mg/kg TOE intragastric daily) for 5 weeks. Behavioral assessments (rotarod, hot plate, cold plate, tail flick) were conducted on day 36 to evaluate sensory and motor functions.

**Figure 2:**
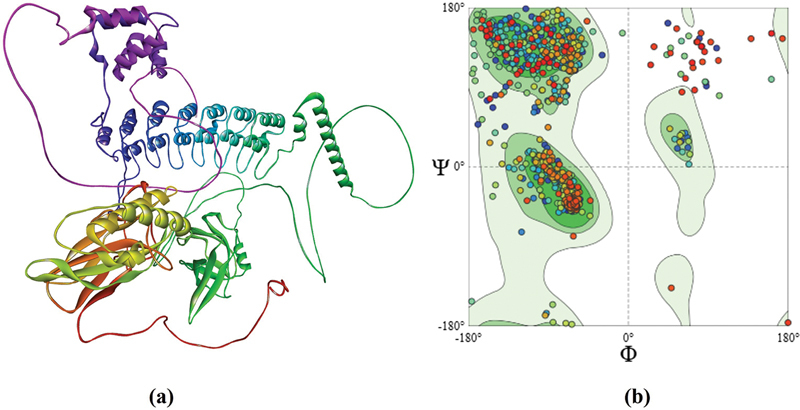
Structural validation of NF-κB1 p105 subunit. Ramachandran plots **(a, b)** generated using SWISS-MODEL for the nuclear factor-kappa-B p105 subunit (P25799, template 1NFK_A). Plot **(a)** shows phi/psi angle distribution, with 92% residues in favored regions, 6% in allowed regions, and 2% in outlier regions, confirming high model quality for docking studies. Plot **(b)** highlights key structural domains.

**Figure 3:**
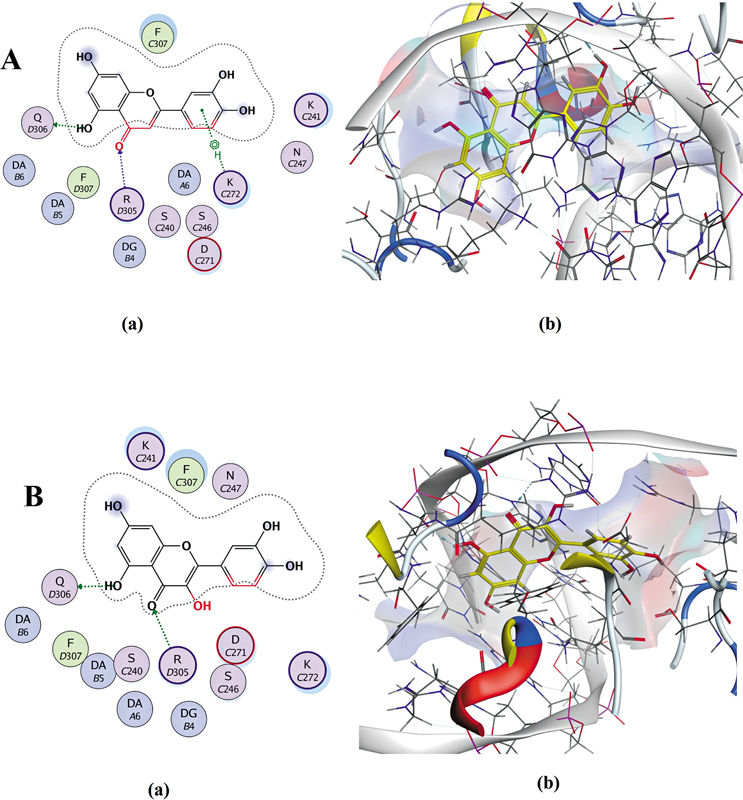
Docking interactions of luteolin **(A)** and quercetin **(B)** with NF-κB1. The 2D and 3D visualizations (PyRx, AutoDock Vina) show hydrogen bonds for luteolin with GLN-306 (2.73 Å), ARG-305 (3.20, 3.46 Å), and a π-H interaction with LYS-272 (4.37 Å), with **(A)** binding energy: -9.11 kcal/mol); and for quercetin with GLN-306 (2.75 Å) and ARG-305 (3.40 Å), with **(B)** binding energy: -8.18 kcal/mol).

**Figure 4:**
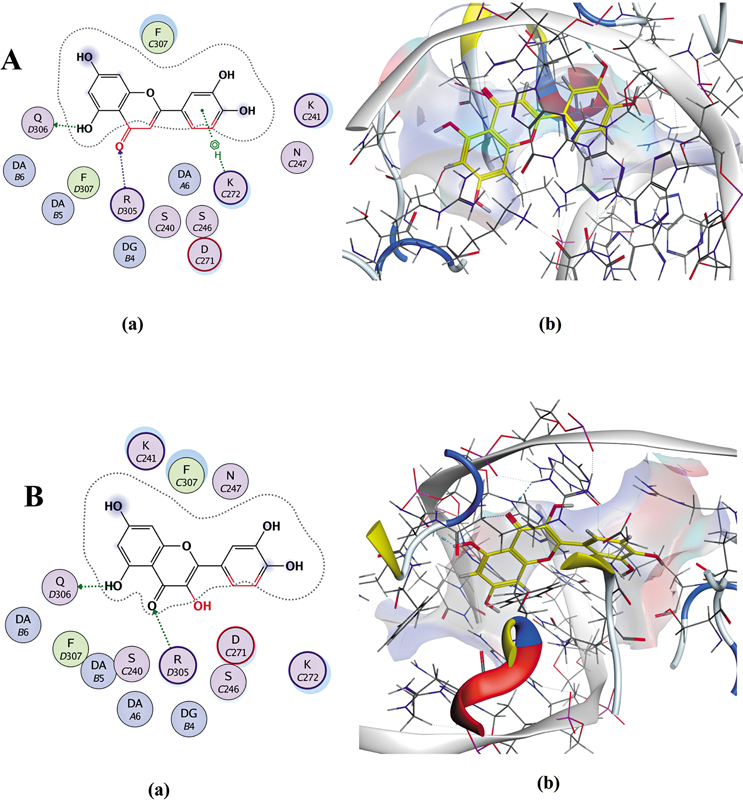
Docking interactions of parthenolide **(A)** and Bay 11-7082 **(B)** with NF-κB1. The 2D and 3D visualizations (PyRx, AutoDock Vina) show a hydrogen bond for parthenolide with LYS-241 (3.05 Å), with **(A)** binding energy: -5.76 kcal/mol); and for Bay 11-7082 with ARG-54 (3.28 Å) and HIS-64 (3.70 Å), with **(B)** binding energy: -5.61 kcal/mol.

Molecular docking ( Figures 3 and 4 ; **Supplementary Material 1 Tables S3A and S3B** ) showed that luteolin and quercetin had the strongest binding affinities (−9.11 and −8.18 kcal/mol, respectively), outperforming synthetic inhibitors parthenolide (−5.76 kcal/mol) and Bay 11-7082 (−5.61 kcal/mol). Luteolin formed hydrogen bonds with GLN-306 and ARG-305, and a π-H interaction with LYS-272 ( Figure 3A ). Quercetin interacted similarly with GLN-306 and ARG-305 ( Figure 3B ). In contrast, synthetic inhibitors exhibited weaker interactions ( Figure 4 ). Other polyphenols like caffeic acid, apigenin, and kaempferol also showed notable affinity (−8.91 to −7.58 kcal/mol), binding to key residues ( **Supplementary Material 2** , available at https://www.arquivosdeneuropsiquiatria.org/wp-content/uploads/2025/09/ANP-2025.0265-Supplementary-Material-2.docx Figure S1 ).

The ADMET analysis ( Table 2 ) confirmed drug-likeness for luteolin and quercetin (MW: 286.24 and 302.24; LogP: 2.28 and 1.99, respectively) with high gastrointestinal absorption and no predicted toxicity. 22 23 These findings supported their selection for in vivo testing.

#### *General observations*

Weekly body weight monitoring showed no significant differences across groups (endpoint mean ± SEM: Control 280 ± 5 g, CIS 265 ± 7 g, TOE + CIS 275 ± 6 g, TOE 285 ± 4 g; *p* > 0.05), indicating no overt toxicity.

### In vivo validation of neuroprotective effects

#### *Behavioral assessments*

Behavioral tests ( Figure 1 ) evaluated sensory and motor function across four groups: Control, TOE, CIS, and TOE + CIS. The CIS group significantly impaired sensory responses (cold plate: +35%, hot plate/tail-flick: +40–50%; *p* < 0.05–0.001) and reduced rotarod performance (−30%, *p* < 0.001). The TOE alone group had no effect, while the TOE + CIS group showed partial recovery: decreased sensory latencies (−20–30%, *p* < 0.05) and improved rotarod scores (+25%, *p* < 0.001), consistent with in silico NF-κB1 inhibition.

#### *Serum inflammatory and oxidative markers*

The use of CIS increased serum NF-κB (+11%, *p* < 0.05), TNF-α (+1.4%), IL-6 (+21%, *p* < 0.001), MDA (+20%), TOS (+21%), and IMA (+11%), while reducing TAS by 25% ( *p* < 0.05), as shown in Table 1 . The group with TOE alone lowered NF-κB (−18%), IL-6 (−29%, *p* < 0.001), MDA (−23%), TOS (−32%), IMA (−13%), and raised TAS by 39% ( *p* < 0.05). The TOE + CIS group showed partial restoration of NF-κB (−12%), IL-6 (−21%), MDA (−21%), TOS (−17%), and TAS (+28%) versus CIS (all *p* < 0.05), indicating reduction of oxidative and inflammatory damage.

#### *Serum antioxidant enzymes*

The CIS group significantly reduced CAT (−4%), GPx1 (−7%), and SOD2 (−8%) ( Table 3 ). The TOE alone group increased CAT (+8%) and GPx1 (+24%), while the TOE + CIS group partially restored CAT (+6%), GPx1 (+12%), and SOD2 (+5%, all *p* < 0.05), confirming its antioxidant-enhancing effects.

**Table 3:** The serum analysis for oxidative stress biomarkers and pro-inflammatory cytokine levels to assess systemic inflammation

Parameters	Baseline (mean ± SEM)	CIS-induced (mean ± SEM)	TOE (500 mg/kg) (mean ± SEM)	CIS + TOE (500 mg/kg) (mean ± SEM)	% Change (TOE + CIS vs. CIS)
**CAT (ng/mL)**	298.51 ± 7.88	285.02 ± 9.74*	308.77 ± 5.46 ^#^	301.08 ± 6.79 ^#^	+5.6%
**Gpx1 (ng/mL)**	189.22 ± 6.58	175.65 ± 5.58*	218.50 ± 12.39 ^##^	196.86 ± 6.01 ^#^	+12.0%
**SOD2 (ng/mL)**	166.99 ± 5.32	153.94 ± 4.35*	173.45 ± 6.30 ^#^	161.95 ± 4.28 ^#^	+5.2%

Abbreviations: CAT, catalase; CIS, cisplatin; SEM, standard error measurement; SOD, superoxide dismutase; TOE, *T. officinale* leaf extract.

Note: Statistical significance: *control vs. CIS, ( *p* < 0.05); ^#^ (CIS vs. TOE + CIS, *p* < 0.05); ^##^ CIS vs. TOE + CIS ( *p* < 0.001).

#### *Sciatic nerve inflammatory and oxidative markers*

In sciatic nerves, CIS elevated NF-κB (+24%, *p* < 0.05), TNF-α (+21%, *p* < 0.001), IL-6 (+40%, *p* < 0.001), MDA (+35%), and TOS (+18%), while reducing TAS by 18% ( *p* < 0.05), as shown in Table 4 . Treatment with TOE alone decreased all inflammatory markers (NF-κB: −21%, TNF-α: −20%, IL-6: −27%) and oxidative stress markers (MDA: −21%, TOS: −30%) and raised TAS (+34%, p < 0.05). The TOE + CIS treatment resulted in significant improvements: NF-κB (−12%), TNF-α (−8%), IL-6 (−16%), MDA (−18%), TOS (−20%), and TAS (+28%, *p* < 0.05), suggesting reduced neuroinflammation.

**Table 4:** The determination of oxidative stress and proinflammatory cytokine profiles in the sciatic nerve to assess localized inflammation

Parameters	Baseline (mean ± SEM)	CIS-induced (mean ± SEM)	TOE (500 mg/kg) (mean ± SEM)	CIS + TOE (500 mg/kg) (mean ± SEM)	% Change (TOE + CIS vs. CIS)
**NF-κB (ng/mL)**	16.17 ± 1.03	19.98 ± 1.47*	15.86 ± 1.92 ^#^	17.68 ± 1.42 ^#^	−11.5%
**TNF-α (ng/L)**	321.70 ± 23.53	388.83 ± 15.74**	309.88 ± 37.76 ^##^	356.00 ± 21.91 ^#^	−8.4%
**IL-6 (ng/L)**	10.31 ± 1.18	14.46 ± 0.79**	10.57 ± 1.51 ^##^	12.13 ± 1.63 ^#^	−16.1%
**IMA (ng/mL)**	122.76 ± 12.10	128.28 ± 13.94	112.99 ± 12.90 ^#^	117.60 ± 8.98 ^#^	−8.3%
**MDA (nmol/mL)**	6.72 ± 0.43	9.06 ± 0.60*	7.14 ± 0.88 ^#^	7.41 ± 0.83 ^#^	−18.2%
**TOS (µmol H₂O₂ Eq./g protein)**	12.12 ± 1.61	14.32 ± 1.69*	10.07 ± 1.54 ^##^	11.42 ± 1.66 ^#^	−20.3%
**TAS (mmol Trolox Eq./g protein)**	1.56 ± 0.21	1.28 ± 0.14*	1.72 ± 0.13 ^#^	1.64 ± 0.22 ^#^	+28.1%
**OSI (TOS/TAS)**	7.77 ± 0.34	11.19 ± 0.16**	5.85 ± 0.39 ^##^	6.96 ± 0.28 ^##^	−37.8%

Abbreviations: CIS, cisplatin; IL-6, interleukin-6; IMA, ischemia-modified albumin; OSI, oxidative stress index; SEM, standard error measurement; MDA, malondialdehyde; NF-κB, nuclear factor-kappa B; TAS, total antioxidant status; TNF-α, tumor necrosis factor-alpha; TOE, *T. officinale* leaf extract; TOS, total antioxidant status.

Note: OSI calculated as TOS/TAS ratio. Statistical significance: *control vs. CIS ( *p* < 0.05); **control vs. CIS ( *p* < 0.001); ^#^ CIS vs. TOE + CIS ( *p* < 0.05); ^##^ CIS vs. TOE + CIS, ( *p* < 0.001).

#### *Sciatic nerve antioxidant enzymes*

The CIS group reduced CAT (−7%), GPx1 (−21%, *p* < 0.001), and SOD2 (−9%) in sciatic nerve tissue ( Table 5 ). The TOE alone group increased CAT (+17%) and GPx1 (+30%), and the TOE + CIS group restored CAT (+27%), GPx1 (+19%), and SOD2 (+18%; *p* < 0.05), demonstrating improved antioxidant defenses in peripheral nerves.

**Table 5:** The measurement of antioxidant levels in sciatic nerve tissue to evaluate oxidative balance

Parameters	Baseline (mean ± SEM)	CIS-induced (mean ± SEM)	TOE (500 mg/kg) (mean ± SEM)	TOE + CIS (500 mg/kg) (mean ± SEM)	% Change (TOE + CIS vs. CIS)
**CAT (ng/mL)**	204.01 ± 33.85	189.60 ± 31.60*	222.57 ± 31.15 ^#^	240.51 ± 36.42 ^#^	+26.8%
**Gpx1 (ng/mL)**	139.15 ± 17.71	109.27 ± 3.87**	142.48 ± 8.04 ^##^	129.54 ± 10.32 ^#^	+18.5%
**SOD2 (ng/mL)**	111.91 ± 6.16	102.28 ± 1.91*	116.44 ± 8.22 ^#^	120.22 ± 2.21 ^#^	+17.5%

Abbreviations: CAT, catalase; CIS, cisplatin; SEM, standard error measurement; SOD, superoxide dismutase; TOE, *T. officinale* leaf extract.

Note: Statistical significance: *control vs. CIS ( *p* < 0.05); **control vs. CIS ( *p* < 0.001); ^#^ CIS vs. TOE + CIS ( *p* < 0.05); ^##^ CIS vs. TOE + CIS ( *p* < 0.001).

#### *Alignment of in silico and in vivo findings*

**Figure 5:**
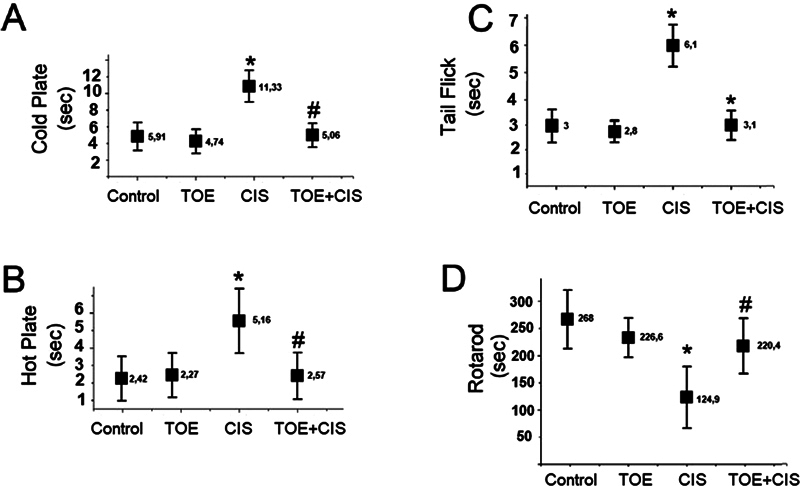
Notes: Statistical significance: *control vs. CIS ( *p* < 0.05), **control vs. CIS ( *p* < 0.001), ^#^ CIS vs. TOE + CIS ( *p* < 0.05), ^##^ CIS vs. TOE + CIS ( *p* < 0.001). Behavioral assessment results. Bar graphs showing latency (seconds) in cold plate (5°C, 50s cut-off), hot plate (55°C, 30s cut-off), tail-flick (10s cut-off), and rotarod (5–40 rpm, 300s cut-off, mean of three trials) tests across four groups: Control (normal saline), TOE (500 mg/kg), CIS (3 mg/kg), and TOE + CIS. Data analyzed by two-way ANOVA with Tukey's post-hoc test.

Luteolin and quercetin, top NF-κB1 inhibitors in silico, effectively reduced inflammatory cytokines and oxidative stress markers in vivo ( Tables 1 and 4 ), while restoring antioxidant enzymes ( Tables 3 and 5 ). Behavioral improvements ( Figure 5 ) paralleled these molecular effects, indicating that TOE's neuroprotective actions stem from modulation of NF-κB1-mediated pathways. The ADMET profiles ( Table 2 ) support bioavailability and nontoxicity, reinforcing therapeutic feasibility.

## DISCUSSION

This study demonstrates that TOE confers robust neuroprotective effects against CIPN in mice models, mediated by potent inhibition of NF-κB1 signaling via its polyphenolic constituents, particularly luteolin and quercetin. These findings address a major therapeutic gap in CIPN management, where existing agents such as duloxetine and gabapentin, provide only partial relief and are associated with significant side effects. 12 13 Through an integrated in silico and in vivo approach, this work provides a comprehensive mechanistic framework supporting TOE as a promising complementary therapy.

Molecular docking analyses revealed that luteolin and quercetin possess superior binding affinities to NF-κB1 (–9.11 and –8.18 kcal/mol, respectively) compared to synthetic inhibitors parthenolide (–5.76 kcal/mol) and Bay 11-7082 (–5.61 kcal/mol). These flavonoids formed stable hydrogen bonds with key residues GLN-306, ARG-305, and LYS-272, indicating strong suppression of NF-κB1-mediated inflammatory signaling. Such molecular interactions are consistent with previous reports demonstrating that flavonoids can inhibit NF-κB activation in neuropathic pain models 13 26 27 and strengthen the biological plausibility of TOE as a safe phytotherapeutic option.

In vivo behavioral assessments supported these computational findings. The TOE therapy significantly ameliorated CIS-induced sensory and motor deficits. The improvements observed in nociceptive tests (tail flick, hot plate, cold plate) and rotarod performance confirm that TOE not only mitigates biochemical damage but also restores functional outcomes relevant to neuropathy management. These results are consistent with evidence that polyphenol-rich extracts alleviate CIPN symptoms through combined antioxidant and antiinflammatory effects. 14 23 24 Additionally, the absence of behavioral changes in the TOE-only group supports its safety and tolerability.

Biochemical findings further demonstrated TOE's neuroprotective activity. The use of CIS markedly increased systemic and peripheral levels of proinflammatory cytokines (NF-κB1, TNF-α, IL-6) and oxidative stress markers (MDA, TOS, IMA), while decreasing antioxidant enzymes (CAT, GPx1, SOD2) and TAS. The use of TOE significantly reversed these changes, lowering proinflammatory and oxidative markers by 12 to 32% ( *p* < 0.05), and restoring antioxidant defenses by 5 to 27% ( *p* < 0.05). These dual actions on oxidative stress and inflammation are likely driven by synergistic effects of multiple polyphenols, highlighting the advantage of plant-based extracts over single-compound strategies. 7 15

The concordance between in silico predictions and in vivo results underscores the reliability of computational docking in guiding experimental studies and therapeutic development. 16 25 27 Moreover, the standardized extract yield (12% w/w), validated by HPLC, enhances reproducibility and clinical relevance. 17 18 Together, these findings suggest that TOE has a translational potential as an adjunctive strategy to reduce the burden of CIPN in oncology patients.

Nevertheless, this study has limitations. First, the use of a single TOE dose limits the assessment of dose-response relationships. Second, sex-specific responses were not evaluated, although emerging evidence suggests gender-based differences in CIPN susceptibility. 23 Third, environmental variability in phytochemical composition necessitates strict standardization for future applications. 17 Fourth, hepatotoxicity was not assessed via liver enzymes, as the study prioritized CIPN neuroprotection and no clinical signs of liver damage were observed, consistent with TOE's known hepatoprotective properties. 10 Fifth, hematological parameters were not evaluated from EDTA plasma, as the focus was on neuroprotective mechanisms in CIPN rather than systemic hematotoxicity. Finally, although ethically justified, the absence of a positive control drug group (such as duloxetine) restricts direct comparison with clinically available agents.

Future research should explore dose-ranging studies, chronic CIPN models, and evaluate TOE's efficacy in combination with other chemotherapeutics. Bioactivity-guided fractionation of TOE could identify the most active polyphenolic components, while mechanistic studies should investigate downstream pathways of NF-κB1 inhibition, including neuronal repair and regeneration. 12 28 Clinical trials utilizing standardized TOE formulations are essential to establish safety and efficacy in human populations.

In conclusion, TOE emerges as a promising, safe, and effective complementary therapy for CIPN. Integrating in silico predictions with in vivo outcomes strengthens its translational value and lays the groundwork for preclinical and clinical investigations aimed at addressing this unmet clinical need.
